# Free sugar profile in cycads

**DOI:** 10.3389/fpls.2014.00526

**Published:** 2014-10-07

**Authors:** Thomas E. Marler, Anders J. Lindström

**Affiliations:** ^1^Western Pacific Tropical Research Center, College of Natural and Applied Sciences, University of GuamMangilao, Guam; ^2^Nong Nooch Tropical Botanical GardenChonburi, Thailand

**Keywords:** accumulation, carbon allocation, cycads, fructose, glucose, maltose, non-structural carbohydrate, sucrose

## Abstract

The sugars fructose, glucose, maltose, and sucrose were quantified in seven tissues of *Zamia muricata* Willd. to determine their distribution throughout various organs of a model cycad species, and in lateral structural roots of 18 cycad species to determine the variation in sugar concentration and composition among species representing every cycad genus. Taproot and lateral structural roots contained more sugars than leaf, stem, female strobilus, or coralloid roots. For example, taproot sugar concentration was 6.4-fold greater than stem sugar concentration. The dominant root sugars were glucose and fructose, and the only detected stem sugar was sucrose. Sucrose also dominated the sugar profile for leaflet and coralloid root tissue, and fructose was the dominant sugar in female strobilus tissue. Maltose was a minor constituent of taproot, leaflet, and female strobilus tissue, but absent in other tissues. The concentration of total free sugars and each of the four sugars did not differ among genera or families. Stoichiometric relationships among the sugars, such as the quotient hexoses/disaccharides, differed among organs and families. Although anecdotal reports on cycad starch have been abundant due to its historical use as human food and the voluminous medical research invested into cycad neurotoxins, this is the first report on the sugar component of the non-structural carbohydrate profile of cycads. Fructose, glucose, and sucrose are abundant in cycad tissues, with their relative abundance highly contrasting among organs. Their importance as forms of carbon storage, messengers of information, or regulators of cycad metabolism have not been determined to date.

## INTRODUCTION

Non-structural carbohydrate (NSC) reserves can be mobilized and deployed to support plant metabolism and growth when current photosynthates are insufficient. This may occur following severe defoliation from events such as herbivory, fire, or tropical cyclone damage, when initial regrowth of foliage and continued maintenance of stem and root tissue depends on stored carbohydrates. A plant’s NSC pool is comprised of low molecular weight sugars (the most abundant free sugars in plants are the disaccharides sucrose and maltose, and the monosaccharides glucose and fructose) plus starch (Chapin et al., 1990). Because of their role in plant resilience in times of stress, they comprise a functional trait that can explain species differences in growth and survival. Therefore, knowledge of the quantity of various NSCs and the relationships among them within various plant organs can improve our understanding of specific growth characteristics and plant responses to seasons and stresses.

Cycads are ancient gymnosperms represented by extant taxa that have retained many primitive features. Their perseverance and ancestral history provides researchers the rare opportunity to gain insight into various aspects of plant evolution and biology (Brenner et al., 2003). The study of cycad taxonomy has received considerable attention, and the result is a description of 300+ species among 10 genera and three families (Osborne et al., 2012). In contrast, the study of cycad horticulture and physiology has been minimal despite the fact that more practical research may shed light on what has enabled persistence of this plant group throughout 100s of millions of years (Norstog and Nicholls, 1997). Moreover, cycads represent the most threatened group of plants worldwide (Hoffmann et al., 2010), so gaining further knowledge regarding all aspects of cycad biology is urgent, and may improve horticultural protocols and aid in developing and implementing successful conservation strategies. Although NSCs undoubtedly play a major role in cycad growth and development, they have not been extensively studied for any cycad species. Because the structural components of cycad stems and roots are primarily living tissue and they have no true wood (Norstog and Nicholls, 1997), reports on NSC relations of woody trees (e.g., Loescher et al., 1990; Nzima et al., 1997) are not reliable for predicting NSC relations of cycads. Furthermore, plants show lineage-specific differences in metabolite composition, but the extent to which the portions of the metabolome can reconcile with cycad taxonomy is unknown.

Our current understanding of the carbohydrate relations of cycad plants is unmistakably incomplete. This work was designed as an initial study to determine the quantity and stoichiometry of four common free sugars among representatives of every described cycad genus, and among various tissues throughout one representative cycad species. We included 18 cycad species growing in a common garden environment. In addition to providing the first look at the distribution and profile of cycad sugars, we addressed several questions. (1) Since the cycad taproot is essentially an extension of the non-woody pachycaulis cycad stem, would stem and taproot tissue exhibit similar amounts and stoichiometry of sugars? (2) Would the tissues with specialized functions such as leaves, strobili, and coralloid roots exhibit amounts and proportional relations of the sugars inconsistent with the structural/storage tissues? (3) Would sucrose dominate the sugar profile in coralloid roots, where cycad endosymbionts carry out nitrogen fixation, in accordance with its dominance in legume nodules?

## MATERIALS AND METHODS

Plants representing 18 species of cycads were sampled from the living collection at the Nong Nooch Tropical Botanical Garden in Chonburi, Thailand. The plants representing many of the species were too valuable to sacrifice for destructive analysis. We therefore evaluated a suitable tissue that could be collected without harming the plants. Sampling leaf tissue would have inflicted the least disturbance to the plants, but we desired the use of storage tissues so leaves were not appropriate for our goals. Stem tissue would have met our goal of storage tissue, but physical damage to a portion of stem tissue elicits extensive secondary damage to cycad stems (Fisher et al., 2009; Marler et al., 2010). Therefore, we could not use stem tissue for the survey. Lateral structural roots were selected as the tissue for analysis, as excision of lateral roots rarely leads to subsequent growth or health problems for cycad plants in horticultural settings. Lateral root tissue was collected from two species for each genus except for the mono-specific *Microcycas calocoma* (Miq.) A. DC. and *Stangeria eriopus* (Kunze) Baill. The species representing the other eight genera were *Bowenia spectabilis* Hook. ex Hook.f., *Bowenia serrulata* (W.Bull) Chamb., *Ceratozamia robusta* Miq., *Ceratozamia miqueliana* H.Wendl., *Cycas machonochiei* Chirgwin and K.D. Hill, *Cycas riuminiana* Porte ex Regel, *Dioon sonorense* (De Luca, Sabato, and Vázq.Torres) J. Chemnick, T.J. Gregory, and S. Salas-Mor., *Dioon spinulosum* Dyer ex Eichl., *Encephalartos mackenziei* L.E. Newton, *Encephalartos laurentianus* De Wild., *Lepidozamia peroffskyana* Regel, *Lepidozamia hopei* (W.Hill) Regel, *Macrozamia macdonnellii* (F. Muell. ex Miq.) A. DC., *Macrozamia mountperriensis* F. M. Bailey, *Zamia encephalartoides* D. W. Stev., and *Zamia muricata.* Species habitat characteristics and plant size were documented (**Table 1**).

**Table 1:** Characteristics of 18 cycad species growing at Nong Nooch Tropical Botanical Garden, Pattaya, Thailand.

Species	Family	Stem height (cm)	Stem diameter (cm)	Native habitat
*Cycas machonochiei*	Cycadaceae	36	19	Tropical
*Cycas riuminiana*	Cycadaceae	124	37	Tropical, wet forest
*Dioon sonorense*	Zamiaceae	22	32	High elevation desert
*Dioon spinulosum*	Zamiaceae	64	24	Lowland tropical rainforest, wet
*Ceratozamia robusta*	Zamiaceae	35	31	Lowland tropical rainforest, wet
*Ceratozamia miqueliana*	Zamiaceae	20	36	Dense rainforest, hot wet summers
*Microcycas calocoma*	Zamiaceae	147	37	Lowland, deciduous forest, dry
*Zamia encephalartoides*	Zamiaceae	39	24	Full sun, hot, montane dry
*Zamia muricata*	Zamiaceae	9	8	Understory, montane forest, wet
*Stangeria eriopus*	Stangeriaceae	16	13	Understory, shade, deciduous forest, dry
*Lepidozamia perrofskyana*	Zamiaceae	72	32	Understory, shade, Lowland rainforest, wet
*Lepidozamia hopei*	Zamiaceae	89	32	Understory, shade, tropical rainforest, wet
*Encephalartos mackenziei*	Zamiaceae	44	42	Full sun, hot, dry
*Encephalartos laurentianus*	Zamiaceae	90	66	Understory, dense forest, wet
*Macrozamia macdonnellii*	Zamiaceae	31	36	Desert, barren rocky slopes
*Macrozamia mountperriensis*	Zamiaceae	15	20	Subtropical, hot wet summers
*Bowenia spectabilis*	Stangeriaceae	18	27	Lowland tropical rainforest, wet
*Bowenia serrulata*	Stangeriaceae	12	12	Understory, shade, tropical rainforest, wet


Stem dimensions are for above-ground portions only.

Female *Zamia muricata* plants were destroyed to use as the model species to determine the general distribution of sugars among various tissues and organs. The plants were 4 years old, supporting female strobili of 8–10 months old. Tissue samples were collected on 19 March 2010 from taproots, lateral structural roots, coralloid roots, stems, petioles, leaflets, and strobili then lyophilized.

Soluble sugar extraction was conducted with hot-water extraction with acetonitrile (80°C; Schloter et al., 2005). The concentrations of sucrose, fructose, glucose, and maltose were determined by HPLC-RI (Thermo Scientific RI-150, AS3000 autosampler, P2000 pump). Our direct and calculated response variables were concentration of the four sugars and total free sugars (sum of the four sugars). In addition, we evaluated the sugar stoichiometry of each tissue category, genus, or family by calculating the quotients among the three dominant sugars: glucose/fructose, glucose/sucrose, and fructose/sucrose. Finally, we determined the relative immediate carbohydrate availability with the quotient hexoses/disaccharides where the hexose content was the sum of glucose and fructose, and the disaccharide content was the sum of sucrose and maltose.

For comparison among tissue categories of *Zamia muricata* plants or among genera or family for the phylogenetic analysis, a one-way ANOVA was performed using Proc GLM function in SAS with Type III *P*-value to determine significance. Means separation when significant was conducted employing the Least Significant Difference test.

## RESULTS

### SUGAR PROFILES AMONG *Zamia muricata* ORGANS

**FIGURE 1:**
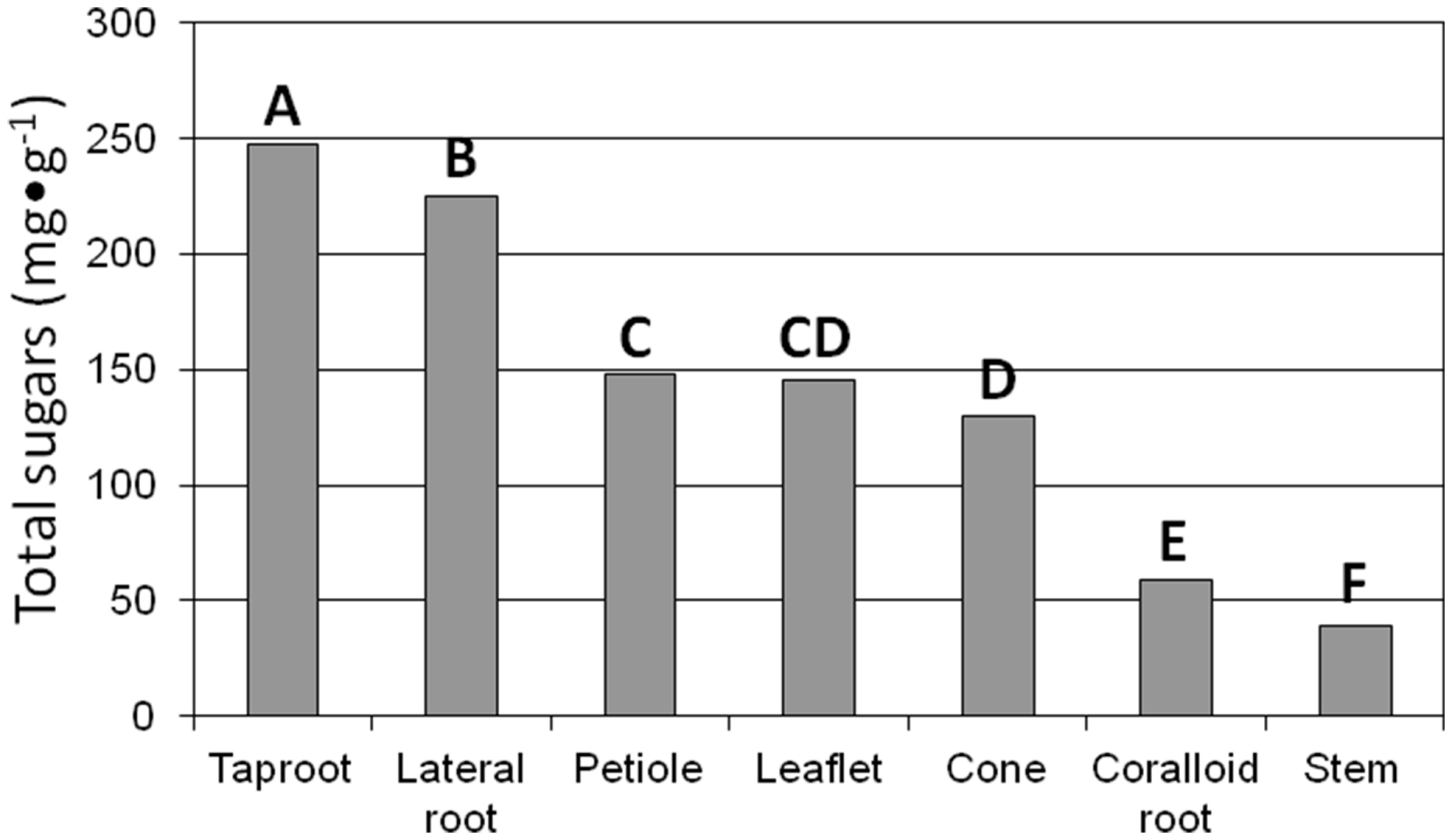
**The influence of tissue of reproductive 4-year-old female *Zamia muricata* plants on total sugar concentration among seven types of tissue.** Means with same letter are not different. *P* < 0.0001.

Total sugar concentration exhibited a 6.4-fold difference (F_6,14_ = 192.39; *P* < 0.0001) among the tissue categories (**Figure 1**). Total sugars ranged from 39 mg⋅g^-1^ for stem tissue to 247 mg⋅g^-1^ for taproot tissue. The sugar concentration in taproot and lateral structural root tissues greatly exceeded that in all other tissue categories. Fructose concentration varied among the tissue categories (F_6,14_ = 226.86; *P* < 0.0001). Fructose was not detected in stem tissue, and ranged from 22 to 108 mg⋅g^-1^ in accordance with coralloid root < leaflet ≤ petiole < strobilus ≤ lateral root < taproot. Glucose concentration exhibited variation among the tissue categories (F_6,14_ = 81.73; *P* < 0.0001). Glucose was not detected in stem or coralloid root tissue, and ranged from 11 to 120 mg⋅g^-1^ throughout the range of leaflet ≤ strobilus < petiole < lateral root < taproot tissues. Sucrose concentration was present in every tissue category yet the range was more constricted than for fructose or glucose. Sucrose concentration varied among the tissue categories (F_6,14_ = 26.56; *P* < 0.0001), ranging from 12 to 87 mg⋅g^-1^ in the order taproot < strobilus ≤ petiole ≤ coralloid root ≤ stem < lateral root < leaflet. Mean maltose concentration was 2.7 mg⋅g^-1^, and maltose did not differ among the tissue categories (F_6,14_ = 2.26; *P* < 0.0985).

**FIGURE 2:**
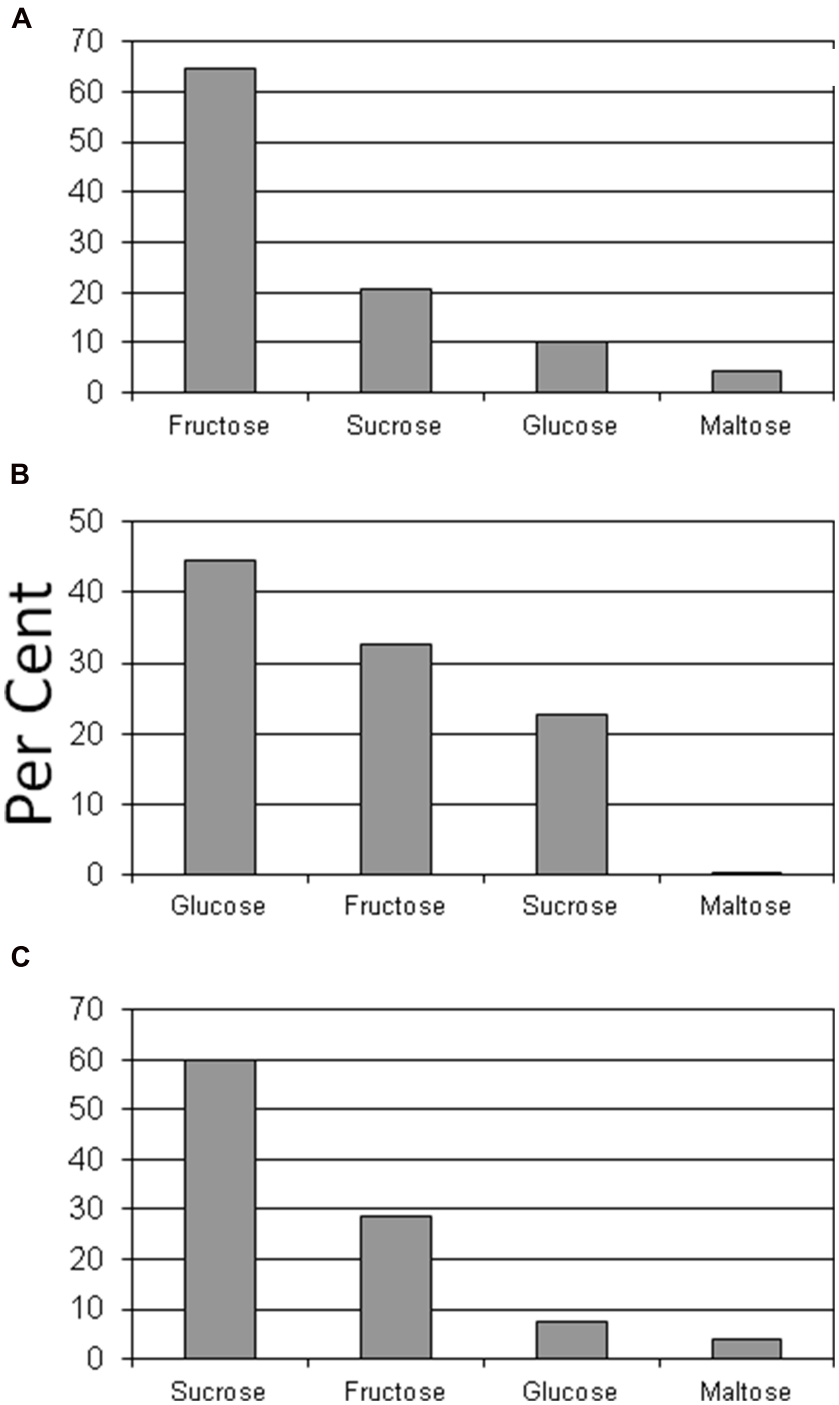
**Per cent of glucose, fructose, sucrose, or maltose comprising total sugar content for female strobilus **(A)**, petiole **(B)**, or leaflet **(C)** tissue of *Zamia muricata* plants**.

**FIGURE 3:**
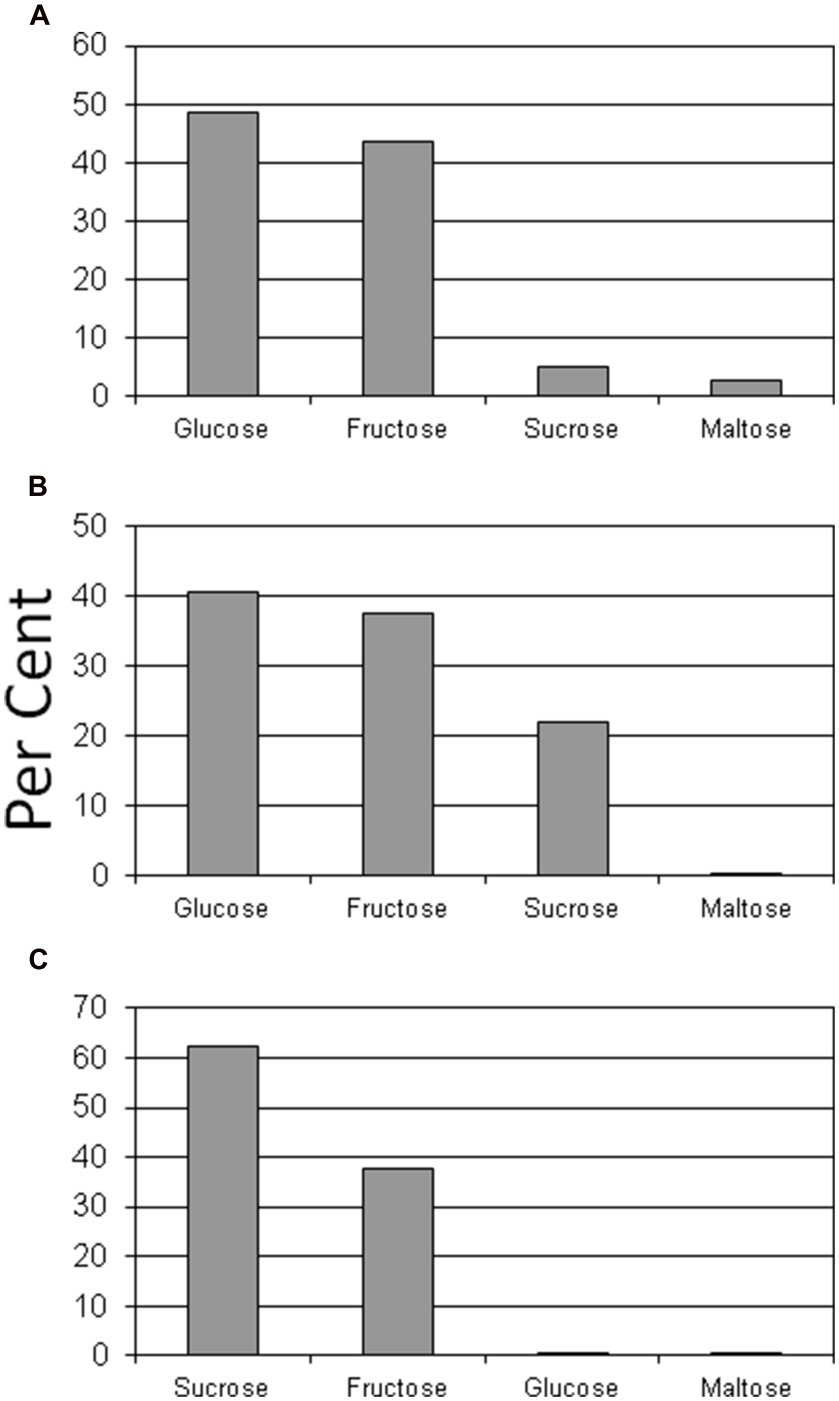
**Per cent of glucose, fructose, sucrose, or maltose comprising total sugar content for taproot **(A)**, lateral structural root **(B)**, or coralloid root **(C)** tissue of *Zamia muricata* plants**.

Glucose and fructose were the dominant sugars in taproots, lateral structural roots, and petioles (**Figures 2** and **3**). Sucrose represented about 60% of the sugars in leaflets and coralloid roots (**Figures 2** and **3**), and was the only sugar detected in stems. Female strobilus tissue was the only tissue in which fructose dominated the sugar profile (**Figure 2**). Sugar diversity was greatest in taproot, leaflet, and strobilus tissues, as these were the only tissues in which all four sugars were detected. Stem tissue was the tissue exhibiting the least diversity in free sugars, as it was the only tissue within which three of the sugars were not detectable.

The quotient glucose/fructose exhibited minimal but significant differences (F_6,14_ = 64.6; *P* < 0.0001) among the tissue categories. Glucose/fructose was <0.3 for coralloid root, female strobilus, and leaflet tissue; and was >1.0 for stem, petiole, lateral root, and taproot tissue. The quotient glucose/sucrose exhibited substantial range (F_6,14_ = 13.61; *P* < 0.0001), with glucose/sucrose in taproot tissue being 10.4 and glucose/sucrose of all other tissue categories being <2.2. The quotient fructose/sucrose differed among the tissue categories (F_6,14_ = 5.12; *P* < 0.0056); and was <1.7 for all tissue categories except for strobilus (6.2) and taproot (9.4). The quotient hexoses/disaccharides significantly differed among the organs (F_6,14_ = 44.89; *P* < 0.0056). This quotient was close to nil for stem, coralloid root, and leaflet tissues; from 3.3 to 3.5 for petiole, lateral root, and strobilus tissue; and 12.2 for taproot tissue.

### SUGAR PROFILES OF STRUCTURAL LATERAL ROOTS AMONG GENERA AND FAMILIES

**FIGURE 4:**
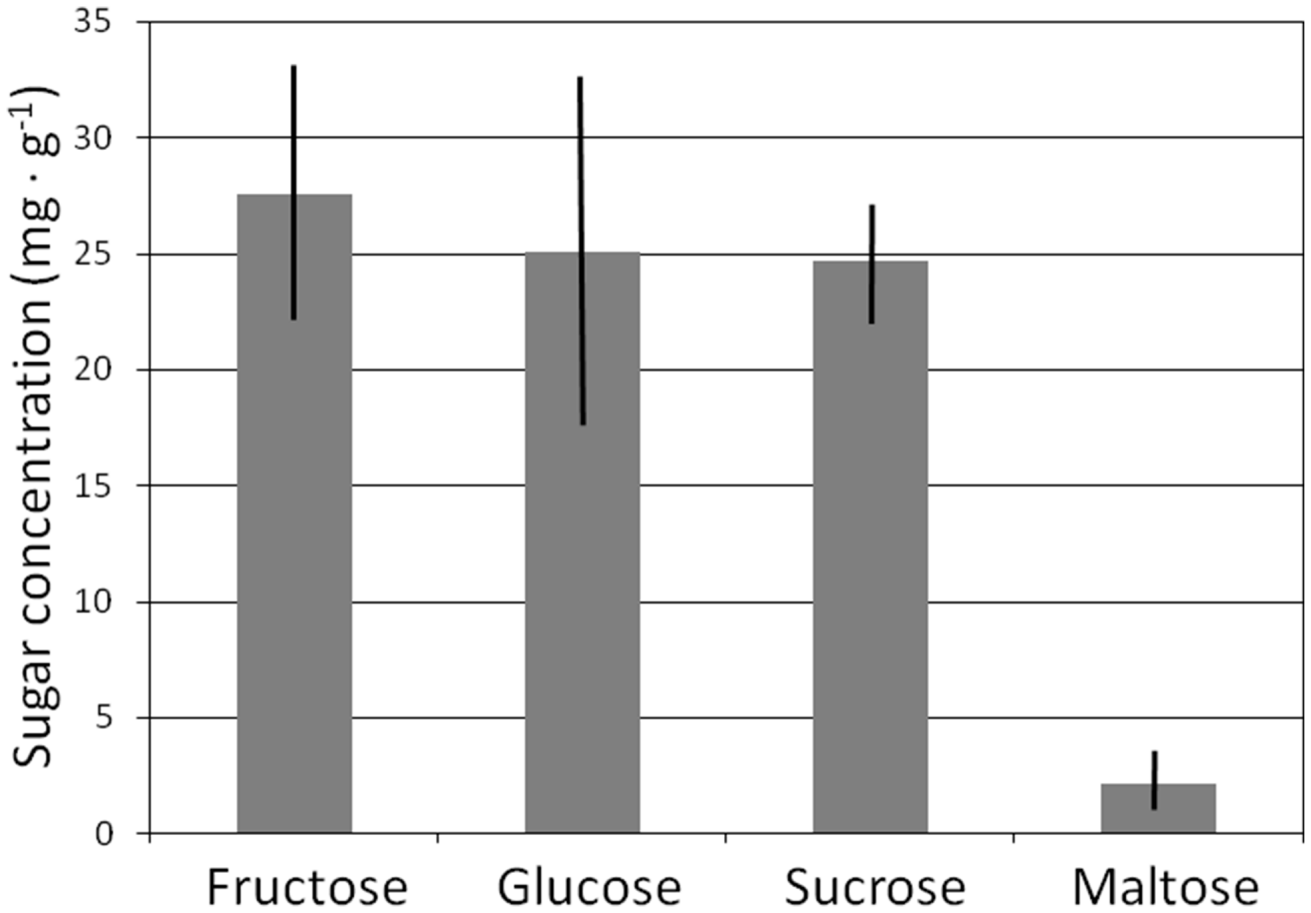
**Mean sugar concentration of lateral structural roots of 18 cycad species.** Mean ± SE.

Total sugar concentration of lateral structural roots exhibited an overall mean of 79.0 mg⋅g^-1^ and did not differ among the 10 genera (*P* < 0.3404) or among the three families (*P* < 0.2073). The total sugar content for these 18 species was fairly evenly split among glucose, sucrose, and fructose fractions (**Figure 4**), and concentration of each of these sugars did not differ among the genera or families. Maltose was a minor component of the lateral root NSC profile, as it was not detected in lateral structural roots of seven genera or one family. Maltose concentration exhibited an overall mean of <10% of that for each of the three dominant sugars (**Figure 4**).

The stoichiometry of these sugars exhibited variables with greater diversity than concentration of each sugar. The quotient glucose/fructose was not influenced by genus (*P* < 0.5676) but differed among families (*P* < 0.0307); and was 0.4 ± 0.2 for Zamiaceae, 1.3 ± 0.3 for Cycadaceae, and 1.7 ± 0.4 for Stangeriaceae. Similarly, glucose/sucrose did not differ among genera (*P* < 0.1366) but differed among families (*P* < 0.0087); exhibiting 0.2 ± 0.2 for Cycadaceae, 0.6 ± 0.3 for Zamiaceae, and 4.4 ± 2.3 for Stangeriaceae. Fructose/sucrose differed among genera (*P* < 0.0028) and families (*P* < 0.0123). Fructose/sucrose was 0.1 ± 0.1 for Cycadaceae, 1.0 ± 0.2 for Zamiaceae, and 2.3 ± 0.7 for Stangeriaceae. The quotient hexoses/disaccharides was not influenced by genera (*P* < 0.1658) but significantly differed among families (*P* < 0.0081). This quotient was 0.4 ± 0.4 for Cycadaceae, 1.4 ± 0.3 for Zamiaceae, and 6.5 ± 3.2 for Stangeriaceae.

## DISCUSSION

Cycad plants are known for long-term survival and resilience (Norstog and Nicholls, 1997), and NSC storage traits may partly explain their ability to cope with ephemeral biotic and abiotic stress. Much has been written about the abundance of starch in cycad tissues (Whiting, 1963; Norstog and Nicholls, 1997; Whitelock, 2002; Williams, 2012). However, the soluble sugar component of NSC relations of cycads has not been reported until now.

The cycad taproot is essentially an extension of the non-woody pachycaulis cycad stem (Marler et al., 2010), so we asked if stem and taproot tissue would exhibit similar amounts of sugars and relationships among sugars. The large storage tissues of stems and taproots of *Zamia muricata* as a representative cycad species were highly contrasting in their sugar relations. In fact, taproots and stems defined the absolute extreme limits of the sugar concentration range when all tissue categories were compared. Taproots contained the greatest while stems contained the least concentration of sugars. Moreover, taproot tissues were highly diverse in sugar type, as all four sugars were represented; while sucrose alone defined the stem tissue sugar signature. Taproots contained more fructose and glucose than any other tissue, yet exhibited less sucrose than all other tissue categories. The sugar profile in stem tissue was highly contrasting from that in taproot tissue. Both organs are primarily comprised of living parenchyma tissue with vascular tissue interspersed throughout. They are essentially the same diameter at the stem-root transition, and are sometimes difficult to distinguish based on external morphology. In stems, the vascular tissue is arranged in organized cylinders, whereas in taproots the vascular tissue is organized in scattered bundles, but in both organs living parenchyma is the major substrate throughout and true wood is absent (Marler et al., 2010). We expected the free sugar characteristics to be similar for these two organ sections that collectively comprise the cycad caudex (Marler et al., 2010), signifying that both would be efficient at NSC storage and serve as potential sites of rapid carbon deployment in times of primary growth. Our results indicated that structural roots alone may be the dominant site of readily available NSC reserves. We note, however, that a full understanding of the respective roles of stem and root source-sink relations will require simultaneous measurement of starch and sugars before, during, and following the ephemeral sink stage of an expensive leaf or strobilus episodic event.

We asked if the tissues with specialized functions such as leaves, strobili, and coralloid roots would exhibit amounts and ratios of the sugars inconsistent with the structural/storage tissues. Indeed, female strobilus tissue was the only tissue type where fructose dominated the sugar profile. Fructose appears to be important in maintenance of *Zamia muricata* female reproductive tissues. The female strobilus was also one of only three tissue categories that exhibited detectable levels of all four sugars. Carbon is generally transported from source to sink organs in higher plants as sucrose (Zimmermann and Ziegler, 1975; ap Rees, 1984). If cycad petioles serve primarily to position leaflet tissues for maximum photosynthesis and as conduits for exported and imported materials, we predicted that the sugars in petiole tissue would primarily be comprised of the sucrose that is exported from leaflet tissue. This was not the case, as glucose and fructose concentrations exceeded those of sucrose in petiole tissue. Perhaps cycad petioles serve a NSC buffering role where various sugars are available for metabolism of nearby leaf or meristem tissues. We also should not discount the possibility that substantial photosynthesis occurs in green petiole tissues, leading to the documented soluble sugar richness. To our knowledge, no measurements of petiole or rachis photosynthesis have been made for any cycad species. Coralloid root structures were positioned directly on large lateral roots, yet the sugar relations were highly contrasting between the two root types. First, glucose concentration in lateral roots was substantial in relation to other tissue categories and in relation to other sugars in lateral roots, yet glucose was not detected in coralloid roots. Second, total sugar concentration of coralloid roots was only 26% of that for lateral roots.

Sucrose is the major sugar represented in legume root nodules that have been studied (Streeter, 1991; Vikman and Vessey, 1993). Hence, whether sucrose would dominate the sugar profile in cycad coralloid roots was of interest. The results with *Zamia muricata* coralloid roots hosting nitrogen-fixing endosymbionts conformed to this legume characteristic, as more than 60% of the sugar profile was represented by sucrose. Cycad-cyanobacteria mutualism occurs in this specialized cycad structure (Grove et al., 1980; Lindblad, 1990; Norstog and Nicholls, 1997; Rai et al., 2000), and the relative abundance of sucrose may indicate its importance in the signaling system that enables success of the mutualism. Further investigations are needed to characterize the role of the endosymbiont as a regulator of coralloid root carbohydrate metabolism (e.g., Rasmussen et al., 2012), a concept that also applies to mycorrhizal relations in cycad roots. Sucrose is synthesized by cyanobacteria, and genes coding for biosynthetic and degradative enzymes have been cloned from cyanobacteria (Lunn, 2002). These traits present unique opportunities to study the carbon metabolism of the coralloid root structure, as both organisms involved in the unique symbiosis are equipped with the tools to catabolize sucrose.

Contemporary cycads represent an ancient lineage of land plants that have commanded the attention of evolutionary biologists (Willis and McElwain, 2002). Resolving the phylogeny of the Cycadales has been challenging (Crisp and Cook, 2011; Nagalingum et al., 2011; Martinez et al., 2012). The Cycadaceae family diverged from the other families as a sister clade in the Triassic or early Jurassic, yet most of the speciation among the three families has occurred in the relatively recent Cenozoic (Nagalingum et al., 2011). We expected to find a diversity in free sugar relations that would align with phylogeny, so the lack of significant differences among the genera and families for most of our response variables indicates remarkable stability in sugar relations among highly contrasting clades that diverged long ago.

The doubt arises as to whether the relationship of each sugar to the other sugars would exhibit variations among genera and families. All cycad genera were expected to contain an abundance of free sugars in structural root tissues due to the abundance of parenchyma in the non-woody construction of all cycad structural tissues (Norstog and Nicholls, 1997). Therefore the stoichiometric relationships among the sugars were expected to exhibit greater variation among the genera than total sugar concentration. Present results confirmed this prediction as the differences in total sugar concentration exhibited no relationship with genus or family. But the quotient hexoses/disaccharides exhibited a significant 22-fold difference among the 10 genera and a significant 17-fold difference among the three families, with the Cycadaceae being much lower than the other families comprising the sister clade.

This study adds to several earlier reports on cycad saccharides (Stephen and De Bruyn, 1967; Siniscalco-Gigliano, 1980, 1990; Moretti et al., 1981; De Luca et al., 1982). Most of the earlier studies were restricted to mucilage chemistry. Moreover, the relationship of cycad taxonomy and secondary metabolites has been discussed [see Richardson (1990) for review], and the collective reports are difficult to interpret. However, many improvements in analytical methods and refining of cycad phylogeny have occurred since these earlier publications.

The present work did not address relevant enzymes or their activities. The regulation of sucrose metabolism by these enzymes has become a central issue in understanding plant carbon relations (Koch, 2004). The biosynthetic enzyme sucrose-phosphate synthase (SPS) plays a major role in controlling sucrose relations (Huber and Huber, 1996). Protein phosphorylation is an essential mechanism controlling SPS activity (Winter and Huber, 2000). Variations in phosphorylation may explain some of the observed differences in *Zamia* photosynthetic and storage organs in the present study. The degradative enzyme sucrose synthase (SUS) participates in starch and sucrose metabolism (Cardini et al., 1955). It catalyzes the reactions that convert fructose and glucose into sucrose, but its functional implications extend well beyond catabolism (Subbaiah et al., 2007). The invertases also cleave sucrose, but the products of the reaction differ from those of SUS and invertase-catalyzed hydrolysis generally has been associated with cell expansion (Winter and Huber, 2000; Koch, 2004).

### APPLICATIONS

Why study cycad sugars? Storage of NSCs enhances plant survival by enabling plants to cope with periods of biotic and abiotic stress. An awareness of NSC relations is therefore needed to fully understand plant susceptibility to and recovery from severe stresses such as drought (Galiano et al., 2011; Muller et al., 2011; Rogiers et al., 2011; Liu et al., 2013; Mitchell et al., 2013), shade (Myers and Kitajima, 2007), fire (Schutz et al., 2009; Wigley et al., 2009), nutrient deficiency (Singh and Sale, 1997; Lei and Liu, 2011; Rellán-Álvarez et al., 2011), pathogens (Angay et al., 2014; Tauzin and Giardina, 2014), and herbivory (Caldwell et al., 1981; Rodgers et al., 1995). Research into all facets of cycad physiology has been minimal to date (Norstog and Nicholls, 1997). Clearly, further research on the impact of biotic and abiotic stresses to cycad populations would benefit from the inclusion of NSC relations and their role as messengers of information. In particular, this is needed to evaluate multiple competing hypotheses that may explain mortality following terminal stress conditions (Fajardo et al., 2011; Adams et al., 2013; Galvez et al., 2013; Keunen et al., 2013; Mitchell et al., 2013).

Brenner et al. (2003) state that physiological roles for cycad metabolites should be investigated and suggested endogenous signaling compounds as a potential line of research. In that light, following are examples that may inform continued cycad NSC research. Efficient regulation of carbon metabolism can give an evolutionary advantage to plants (Feugier and Satake, 2013) and sugars directly regulate various physiological and developmental events as signaling molecules (Rolland et al., 2006; Tognetti et al., 2013). There is growing evidence that sugars exert a regulatory influence over senescence (O’Grady et al., 2013; Thomas, 2013). Sucrose is a direct signal during regulation of fruit ripening (Jia et al., 2013). It also controls the expression of genes involved in starch or fructan synthesis (Cairns and Pollock, 1988; Müller-Röber et al., 1990) and those involved in photosynthesis (Sheen, 1990; Krapp et al., 1991; Van Oosten and Besford, 1994). Similarly, sucrose or glucose alone can replace light as the trigger to up-regulate nitrate reductase gene expression (Cheng et al., 1992). Price et al. (2004) reported that almost a thousand *Arabidopsis* genes were up- or down-regulated by glucose [see Halford et al. (2011) for related review]. Moreover, Matsoukas et al. (2013) report that the interplay between starch and sugars is involved in inciting the transition from juvenile to adult phases in *Arabidopsis*. Recent evidence illuminates that sugars play a central role as the initial regulator of apical dominance rather than auxins as indicated by conventional wisdom (Mason et al., 2014; Van den Ende, 2014).

Relations among starch and various sugars can change with organ development and with season (Boldingh et al., 2000). For example, Nzima et al. (1997) reported that pistachio trees that begin the growing season with greater NSCs produce copious fruit loads, then end the season with less NSC reserves than trees that produce less fruit load. Similarly, initial growth and early yield of strawberry transplants were correlated with pre-transplant carbohydrate status (Kirschbaum et al., 1998; Palha et al., 2002). If similar relations apply to cycads, then success of transplanting or sucker removal for propagation may be influenced by timing of the operation in relation to recent plant developmental and seasonal events that directly influence ephemeral availability of NSCs.

Cycad growth and development is characterized by episodes of rapid leaf or strobilus expansion, followed by longer periods of “rest” during which no apparent primary growth occurs (Marler and Dongol, 2011). This form of plant behavior may be regulated by stored carbon reserves, as endogenous NSCs have been linked to the control of episodic growth in other species (Kuehny et al., 1997; Li et al., 1998). The number of leaf flushes per year and the interval between successive organ expansion events are highly contrasting among cycad species, even when grown in a common garden set up.

The assimilation products produced by photosynthesis are resource revenues that may serve as inputs to resource budget models that inform timing and magnitude of plant reproduction (Isagi et al., 1997). Indeed, the cost of reproduction has been demonstrated for cycads by documenting frequency of reproductive episodes, and the costs associated with production of a female strobili is consistently greater than with production of a male strobili (Clark and Clark, 1987, 1988; Ornduff, 1987; Clark et al., 1992; Marler, 2010). Mechanisms that control these differences may be explained by NSC depletion during and NSC recovery following a growth episode. Therefore, studies that determine pools of NSCs in cycad roots and stems before a leaf or strobilus growth event versus immediately following full expansion of that event would improve our understanding of which organs and their stored metabolites are involved in supporting the considerable biomass additions involved in cycad plant growth events.

Observations of cycad plant mortality following the development of a strobilus are common, especially when this occurs on young or undersized female plants (personal observations), providing indirect evidence of carbon starvation as a result of strobilus development. Therefore, a more thorough understanding of which metabolites and organs are involved as sources may aid in understanding this unfortunate feature of cycad growth and development, and lead to *ex situ* management decisions that may mitigate these outcomes.

Maltose is the disaccharide that emerges from the breakdown of starch (Lloyd et al., 2005). Maltose has been proposed to confer protection against degradation of PSII, and the suppression of maltose production results in decreased PSII photochemical efficiency (Kaplan and Guy, 2005). The modest maltose levels in leaflets and female strobili, which were presumed to be photosynthetic, may serve this function.

## CONCLUSION

The implications of this initial look at free sugars in cycads are far-reaching and provide novel insights. The diversity of free sugars and their elevated concentrations in cycad roots illuminates a sharp contrast to the paucity in sugar diversity and muted concentration in the pachycaulis stems. The stoichiometry of free sugars was more influenced by phylogeny than was absolute concentrations. For example, hexoses/disaccharides of Cycadaceae was a fraction of that for the other families collectively representing a sister clade. These traits may be of importance to ecologists for understanding plant behavior in natural habitats, and to conservationists of rare taxa for informing horticultural management decisions. The present work leaves several unresolved issues for future work. For example, the influence of sink activity of primary growth episodes on pools of free sugars among storage organs is unknown. The influence of free sugar pools on biotic and abiotic stress relations is also unknown for any cycad species. The role of free sugars in plant signaling represents a research priority that is yet to be explored (Tognetti et al., 2013), and the study of cycad sugar relations may add greatly to this agenda. At least one cycad species has been shown to possess crassulacean acid metabolism in leaf photosynthesis (Vovides et al., 2002), and the antiquity of cycads may offer a unique look at evolution of carbon concentrating mechanisms in plants (Raven et al., 2008; Reinert and Blankenship, 2010). The role of relevant enzymes in sucrose metabolism were not addressed in this study. The underlying physiological reasons for the observed sugar profiles may be more fully understood with direct research on enzyme activities in addition to metabolite levels. Finally, functional aspects of NSCs in cycads may be more fully understood by studying ecological correlates among species grouped by ecotype rather than by studying phylogenetic patterns among species grouped by taxonomy.

## Conflict of Interest Statement

The authors declare that the research was conducted in the absence of any commercial or financial relationships that could be construed as a potential conflict of interest.
